# Membranous nephropathy with solitary polyclonal IgA deposition: A case report and literature review 

**DOI:** 10.5414/CNCS109807

**Published:** 2019-10-28

**Authors:** Masato Sawamura, Atsushi Komatsuda, Hajime Kaga, Ayano Saito, Tadashi Yasuda, Hideki Wakui, Kensuke Joh, Naoto Takahashi

**Affiliations:** 1 Department of Hematology, Nephrology, and Rheumatology, Akita University Graduate School of Medicine,; 2 Department of Internal Medicine, Honjo Daiichi Hospital,; 3 Department of Life Science, Akita University Graduate School of Engineering Science, Akita, and; 4 Department of Pathology, Jikei University School of Medicine, Tokyo, Japan

**Keywords:** membranous nephropathy, rare disease, solitary polyclonal IgA deposition

## Abstract

A 60-year-old man presented with nephrotic syndrome (NS). Light microscopy of renal biopsy specimens showed minor glomerular abnormalities, while immunofluorescence microscopy revealed solitary polyclonal granular IgA deposition along the glomerular capillary walls. Electron microscopy showed small amounts of electron-dense deposits in the subepithelial area, but not in the mesangial area. In this patient, apparent underlying disease was not found during the 3-year follow-up, and low-dose prednisolone was effective in the treatment of NS. To our knowledge, there is only one case report of membranous nephropathy with clinicopathological features similar to our case.

## Introduction 

Membranous nephropathy (MN) is one of the most common causes of nephrotic syndrome (NS) in adults [1]. Approximately 80% of cases are renal limited (primary MN: PMN) and 20% are associated with other systemic diseases or drug exposure (secondary MN: SMN). Most cases of PMN have circulating IgG4 autoantibodies to the M-type phospholipase A2 receptor (PLA2R) (70%) or antibodies to the thrombospondin type-1 domain-containing 7A (3 – 5%) [1]. Based on these immunological features, PMN is now classified as an IgG4 autoimmune disease [2]. The recognition that PMN is an autoimmune disease has markedly altered both the diagnostic and therapeutic approaches to what was previously called idiopathic MN [1]. 

Immunofluorescence microscopic findings of PMN are characterized by granular staining for IgG, predominantly IgG4, along the glomerular capillary walls [1]. Electron microscopy of PMN confirms the exclusively subepithelial localization of electron-dense deposits [1]. Biopsy findings that should prompt careful search for SMN include dominant depositions of IgG1/IgG3, IgA, IgM, or C1q, and electron-dense deposits in subendothelial or mesangial locations [1]. To our knowledge, there are several case reports of monoclonal IgA-type MN or IgA-type monoclonal immunoglobulin deposition disease (MIDD) with membranous features [3, 4, 5] and only one case report of MN with solitary polyclonal IgA deposition [6]. Here, we report an additional case of MN with solitary polyclonal IgA deposition. 

## Case report 

A 60-year-old Japanese man was admitted to Honjo Daiichi Hospital due to proteinuria and edema. He had been diagnosed with type 2 diabetes. On admission, his blood pressure was 136/83 mmHg. A physical examination showed bilateral pretibial edema. No abnormal signs were observed in the lungs, heart, or abdomen. 

Urinalysis showed heavy proteinuria (5.1 g/g creatinine) without hematuria. His leukocyte count, hemoglobin level, and platelet count were 5,900/µL, 14.7 g/dL, and 191,000/µL, respectively. Serum total protein was 5.2 g/dL, albumin 2.4 g/dL, blood urea nitrogen 8.4 mg/dL, creatinine 0.79 mg/dL, alanine aminotransferase 16 U/L, aspartate aminotransferase 11 U/L, lactate dehydrogenase 206 U/L, total cholesterol 261 mg/dL, IgG 572 mg/dL, IgA 345 mg/dL, IgM 93 mg/dL, C3 80 mg/dL (normal range: 65 – 135 mg/dL), and C4 12 mg/dL (normal range: 13 – 35 mg/dL). Tests for anti-nuclear antibodies, cryoglobulin, hepatitis B virus antigen, and anti-hepatitis C antibodies were all negative. Monoclonal proteins were not detected in the serum or urine. Circulating IgA-class anti-PLA2R antibodies were not determined by our in-house ELISA [7] using peroxidase-conjugated anti-human IgA as a secondary antibody. 

**Figure 1.**
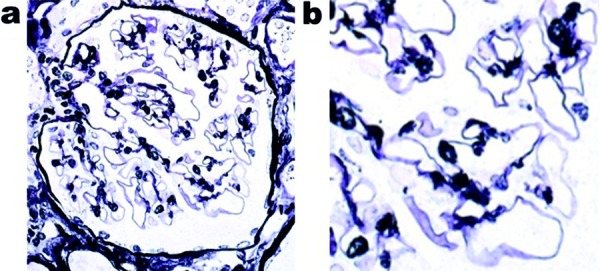
a, b: Light microscopy shows no spike formation or bubbling in the glomerular capillary wall (periodic acid-methenamine-silver staining × 400). b: Enlarged image.

**Figure 2.**
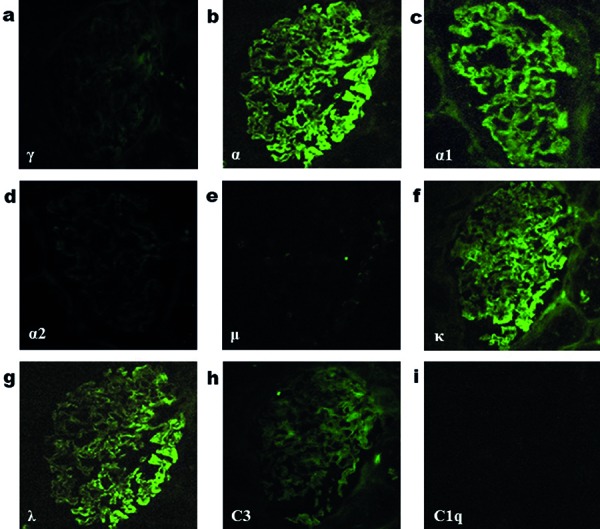
Immunofluorescence staining for (a) IgG (γ-heavy chain); b: IgA (α-heavy chain); c: IgA1 (α1-heavy chain); d: IgA2 (α2-heavy chain); e: IgM (µ-heavy chain); f: κ-light chain; g: λ-light chain; h: C3; i: C1q.

**Figure 3.**
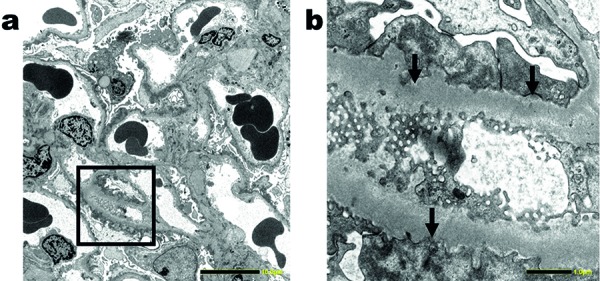
a: Electron microscopy shows extensive foot process effacement of the podocytes, which contain large amounts of electron-dense materials mainly in the area covering the surface of the glomerular basement membrane. Bar = 10 µm. b: In higher magnification (inside the black square of Figure 3a), small amounts of electron-dense deposits are seen beneath the cytoplasm of the podocytes containing the dense materials (arrows). Bar = 1 µm.

Due to the presence of NS, a renal biopsy was performed. Light microscopy showed global scleroses in 2 of 20 glomeruli. The functioning glomeruli showed no mesangial proliferation and no bubbling/spike appearance along the glomerular capillary walls (Figure 1). In the tubulointerstitium, mild lymphocyte infiltration, tubular atrophy, and interstitial fibrosis were observed. There was moderate arteriolar hyalinosis. Immunofluorescence microscopy showed 2+ granular staining for IgA- and IgA1-heavy chains (Figure 2b, c), 2+ granular staining for κ- and λ-light chains (Figure 2f, g), and trace granular staining for C3 (Figure 2h) along the glomerular capillary walls, but no significant staining for IgG-, IgA2-, IgM-heavy chains, or C1q (Figure 2a, d, e, i). An immunofluorescence study using anti-PLA2R antibodies (Sigma-Aldrich, St. Louis, MO, USA) showed negative glomerular staining. Electron microscopy revealed extensive foot process effacement of the podocytes, which contained large amounts of dense materials mainly in the area covering the surface of the glomerular basement membrane, but not in the mesangial area (Figure 3a). Small amounts of electron-dense deposits were observed beneath the cytoplasm of the podocytes containing the dense materials (Figure 3b). From the above-mentioned pathological findings, the diagnosis of early stage MN with solitary polyclonal IgA deposition was made. 

He was treated with low-dose prednisolone (PSL) (10 mg/day for 8 weeks) in consideration of his history of type 2 diabetes. Thereafter, partial effects on proteinuria were observed, and PSL doses were gradually tapered. At the 3-year follow-up he was well. His urinary protein was 2.1 g/g creatinine, serum total protein 5.8 g/dL, albumin 3.5 g/dL, and creatinine 0.87 mg/dL. 

## Discussion 

In the present study, we reported a patient with NS caused by solitary polyclonal IgA1 deposition along the glomerular capillary walls. Electron microscopy revealed small amounts of electron-dense deposits in the subepithelial area, but not in the mesangial area. These findings were consistent with those of early-stage MN. In this patient, apparent underlying disease was not found during the 3-year follow-up, and low-dose PSL was effective for the treatment of NS. 

There are several variants of glomerular disease with IgA deposits combined with capillary wall abnormalities. IgA-dominant postinfectious glomerulonephritis sometimes has conspicuous subepithelial dense deposits, although these often have a hump appearance [8]. However, there was no evidence for a concurrent infection, or a pattern of injury suggestive for IgA-dominant postinfectious glomerulonephritis in our patient. 

Coexistence of IgA nephropathy (IgAN) and MN is rarely seen in the same patient. In Chen et al.’s [9] series of IgAN, 26 out of 3,543 patients (0.7%) had combined IgAN-MN. Mesangial expansion and mesangial hypercellularity, which are characteristic findings of IgAN [10], were observed in all these patients. The proportion of circulating IgG-type anti-PLA2R antibodies in patients with IgA-MN, detected by immunofluorescence assay using HEK293 cells (Euroimmun, Lübeck, Germany), was significantly lower than that of primary MN patients in their cohort. In their study, none of the patients with IgA-MN was diagnosed with SMN. They speculated that the occurrence of superimposed MN combined with a background of preexisting IgAN causes combined IgAN-MN. However, this entity is different from our case, because it has capillary wall IgG immune deposits combined with mesangial IgA-dominant deposits [9]. 

There are several case reports of monoclonal IgA-type MN or IgA-type MIDD with membranous features [3, 4, 5]. Sethi [3] reported an MN patient with IgA-κ deposits and crescents. This case was considered to be secondary to underlying monoclonal gammopathy. Miura et al. [4] reported a patient with IgA1-λ-type MIDD associated with membranous features, a rare type of MIDD [11]. In this case, the relationship between chronic hepatitis C viral infection and monoclonal gammopathy was considered. Kitazawa et al. [5] reported a case of IgA1-λ-type MIDD associated with membranous features. This case also had IgG4-related tubulointerstitial nephritis. 

To the best of our knowledge, only one case of solitary polyclonal IgA deposition was reported, namely by Kobayashi et al. [6] in 2015. This patient and ours did not show mesangial IgA deposition, which is a characteristic finding of IgAN [10]. Therefore, pathological features in these two cases are not consistent with those of combined IgAN-MN. Table 1 summarizes clinicopathological findings in the previously reported case [6] and the present case. The patients were a 71-year-old Japanese female and a 60-year-old Japanese male. Both patients developed NS with mild hematuria or without hematuria. Renal function was preserved. Normocytic anemia was found in the previously reported case, while the level of serum C4 was slightly low in the present case. Anti-nuclear antibodies, cryoglobulin, or monoclonal proteins were not detected. Low- or medium-dose PSL therapy was effective for treatment of NS. During the follow-up period of 3 – 8 years, no definitive cause, such as malignancy or infection, was identified. Renal biopsy findings were consistent with those of stage I – II MN or early stage without proliferative changes. Solitary polyclonal IgA deposition (IgA1 deposition in the present case) was a characteristic immunofluorescence finding, while complement deposition was negative or at trace levels. 

The mechanisms of MN with solitary polyclonal IgA deposition in the previously reported case and the present case are unknown. In the present case, circulating IgA-class anti-PLA2R antibodies were not detected by our in-house ELISA [7]. Other known autoantigens in patients with primary MN [1] or exogenous mucosal antigens, such as bacterial antigens, may be associated with solitary polyclonal IgA deposition with MN features. 

In conclusion, MN associated with solitary polyclonal IgA deposition is an extremely rare entity. Study of similar cases is needed for further determination of its clinicopathological features and outcome. 

## Funding 

None. 

## Conflict of interest 

The authors declared no conflict of interest. 

**Table 1.** Clinicopathological findings of MN with solitary polyclonal IgA deposition in the previously reported case and the present case.

	Reported case [6]	Present case
Age (years)	71	60
Gender	Female	Male
Hypertension	(+)	(–)
Edema	(+)	(+)
Complication		Type 2 diabetes
Proteinuria (g/day or g/g creatinine)	4.8	5.1
microscopic hematuria (> 5 RBC/HPF)	(+)	(–)
Serum albumin (g/dL)	2.1	2.4
Serum creatinine (mg/dL)	0.8	0.79
White blood cell (/µL)	ND	5,900
Hemoglobin (g/dL)	10.3	14.7
Platelet (/µL)	ND	191,000
Serum C3 (mg/dL)	147	80
Serum C4 (mg/dL)	32	12
Serum anti-nuclear antibody	(–)	(–)
Serum cryoglobulin	ND	(–)
Serum IgG (mg/dL)	1,030	572
Serum IgA (mg/dL)	271	345
Serum IgM (mg/dL)	ND	93
Monoclonal protein		
Serum	(–)	(–)
Urine	(–)	(–)
Treatment (initial dose of PSL)	PSL (25 mg/day)	PSL (10 mg/day)
Follow-up period (year)	8	3
Proteinuria (g/day or g/g creatinine) at follow-up	2	2.1
Serum creatinine (mg/dL) at follow-up	ND	0.87
Light microscopy		
No. of glomeruli	12	20
No. of sclerosis	1	2
GBM thickening	(+)	(–)
Bubbling/spike appearance	(+)	(–)
Mesangial proliferation	(–)	(–)
Interstitial lymphocyte infiltration	ND	Mild
Tubular atrophy	ND	Mild
Interstitial fibrosis	ND	Mild
Vascular alterations	ND	Moderate
Immunofluorescence microscopy		
IgG	(–)	(–)
IgA	(+)	IgA1/IgA2 (+)/(–)
IgM	(–)	(–)
κ/λ	(+)/(+)	(+)/(+)
C3	(–)	Trace-positive
C1q	(–)	(–)
Electron microscopy		
Subepithelial granular deposits (MN stage)	(+) (I to II)	(+) (early)
Subendothelial granular deposits	(–)	(–)
Mesangial granular deposits	(–)	(–)

GBM = glomerular basement membrane; HPF = high-power field; MN = membranous nephropathy; ND = not described; PSL = prednisolone; RBC = red blood cells.
